# Structural and Thermal Properties of Montmorillonite/Ionic Liquid Composites

**DOI:** 10.3390/ma12162578

**Published:** 2019-08-13

**Authors:** Olga Alekseeva, Andrew Noskov, Elena Grishina, Lyudmila Ramenskaya, Nadezhda Kudryakova, Vladimir Ivanov, Alexander Agafonov

**Affiliations:** 1 G.A. Krestov Institute of Solution Chemistry, Russian Academy of Sciences, Ivanovo 153045, Russia; 2 Kurnakov Institute of General and Inorganic Chemistry, Russian Academy of Sciences, Moscow 117901, Russia; 3 National Research Tomsk State University, Tomsk 634050, Russia

**Keywords:** ionic liquid, montmorillonite, porosity, glass transition temperature, thermal stability, FTIR spectra

## Abstract

Composites of montmorillonite K10 (MMT K10) and ionic liquid (IL) containing a 1-butyl-3-methyl-imidazolium cation ([BMIm]^+^) and various anions, such as bis (trifluoromethylsulfonyl) imide ([NTf_2_]^−^), trifluoromethanesulfonate ([OTf]^−^), and dicyanamide ([DCA]^−^) have been obtained in this work. A number of methods, such as dynamic light scattering (DLS), scanning electron microscopy (SEM), X-ray diffraction (XRD), thermal gravimetry (TG), differential scanning calorimetry (DSC), Fourier-transform infrared (FTIR) spectroscopy, and nitrogen adsorption–desorption have been used to characterize clay, and to study the structure and thermal behaviour of the composites. It has been found that the MMT K10 powder has a narrow particle size distribution with a peak at 246 nm and a mesoporous structure (S_BET_=195 m^2^/g). According to the FTIR spectra, MMT K10/IL interaction depends on the IL type. It has been identified that confined ionic liquid interacts with both clay and adsorbed water in accordance with the hydrophilicity and size of the anion, in the following order: [DCA]^−^ > [OTf]^−^ > [NTf_2_]^−^. Characteristic temperatures of glass transition, crystallization, and melting have been determined for the ionic liquids under study and their MMT K10 composites. It has been revealed that when IL is adsorbed on the surface of clay, the phase transitions in IL change. The greatest changes are observed in the case of BMImNTf_2_. By applying the method of thermogravimetric analysis, it is shown that composite formation is accompanied by a decrease in the IL thermal stability. Apparently, the highly developed surface of montmorillonite K10, obtained by acid treatment, plays a major role in the decrease in the IL’s thermal stability. The influence of the IL anion on the thermal and spectral characteristics of an MMT K10/IL composite was studied for the first time.

## 1. Introduction

Increasing attention has recently been paid to the development of functional materials based on clay minerals, found in abundance around the world, due to their use in various industries. The morphology and physico-chemical properties of clay minerals are determined by their high specific surface area and presence of exchangeable cations, hydroxyl groups, and adsorbed water molecules in their structure.

There are a lot of kinds of clays, such as smectite, kaolinite, vermiculite, etc. [1]. Among them, montmorillonite (MMT) is a natural and cost-effective clay that belongs to the structural family known as the 2:1 phyllosilicates, consisting of a tetrahedral sheet sandwiched between two parallel octahedral sheets [2,3,4]. Stacking of these layers generates van der Waals gaps or “galleries”. The galleries are occupied by inorganic cations, such as Na^+^, Ca^2+^, and Mg^2+^. Due to its high surface area, cation intercalation, and excellent thermal stability, MMT is a material of great interest to researchers working in areas related to adsorption and catalysis, as well as the development of sensors, filtration membranes, and membranes for Li-ion batteries [5].

Interlayer cations in MMT are capable of ion exchange, and can be replaced by other inorganic or organic ions, metal nanoparticles, metal oxides, or metal–organic compounds [6,7,8]. This allows for the creation of composite nanomaterials with new properties, such as photocatalytic, catalytic, adsorption, mechanical, and thermal stability.

Recently, there has been interest in nanocomposites of montmorillonite with ionic liquids [9,10]. This composite material retains the chemical inertness, low vapor pressure, and high electrical conductivity inherent in ionic liquids, and significantly reduces its fluidity. In addition, an expensive component can be replaced by a cheaper one, while maintaining the unique properties of ionic liquids. In some cases, interlayer cations in montmorillonite can be replaced by inorganic or organic cations from ionic liquids [11].

The modification of clay minerals opens up the opportunity for obtaining new materials with special properties, as well as potential technical and environmental applications. The use of ionic liquids in MMT modification has drawn attention because the ionic exchange with these compounds allows an easy organic modification in clay minerals.

Ionic liquids are a new class of solvents (green solvents): salts that are liquid at room temperature, consisting of organic cations and organic or inorganic anions. Due to their unique characteristics, such as low volatility, non-flammability, high thermal stability (up to 400 °C), and high ionic conductivity, ILs can be used in many important applications, such as catalysis and extraction, as well as electrolytes in electrical devices (e.g., batteries, capacitors, fuel cells, and solar cells) and in different electrochemical processes (e.g., the dissolution of metals, coating, and polishing) [12,13,14,15]. For the successful resolution of electrochemical issues, theoretical studies of ILs are required. In this regard, it is necessary to mention the work of Fedorov and Kornyshev [16,17], in which the authors performed molecular dynamics simulations and analyzed the structure of the double layer for some ionic liquid models.

Ionic liquids can be produced with a variety of cations and anions. Most of the ILs used for MMT modification have cations based on imidazolium, pyridinium, and pyrrolidinium ions [18,19]. Certainly, the presence of IL organic cations in the MMT structure increases the hydrophobicity of the mineral clay, and makes it suitable for absorbing organic substances as pollutants [11,20].

As a result of interaction with clays, ionic liquids change fluidity, the temperature interval of the liquid state, and thermal stability, while maintaining electrical conductivity. The physical and chemical properties of such composites depend on the interaction of the ionic liquid with the porous space of the clay. In this case, due to the confinement effect, the properties of both the inorganic component and the ionic liquid change, which leads to a change in the characteristics of the resulting material. The interactions in such systems are difficult to predict a priori, since the properties of the obtaining materials are influenced by many factors related both to the features of the clay nanostructure and the properties of ionic liquids. The mechanisms of interactions between ILs, clays, and the interlayer configurations of ILs are still debated [21].

In present work, the montmorillonite K10 was modified by ionic liquids, which have a 1-butyl-3-methylimidazolium cation with various anions: bis (trifluoromethylsulfonyl) imide, trifluoromethanesulfonate, or dicyanamide.

MMT K10 is an acid-activated clay, in which the edges of crystals open during acid treatment and the octahedral cations (Al or Mg) are leached from the MMT structure. This results to the gradual destruction of the octahedral layers and an increase in surface area to 220–270 m^2^/g, whereas for natural clay this value is near 20 m^2^/g [22].

**Figure 1:**
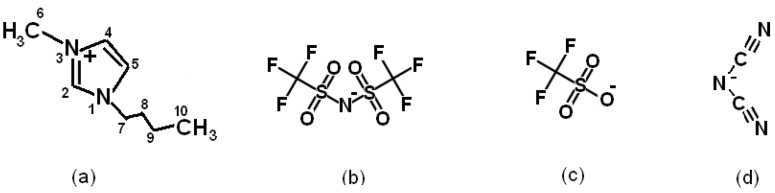
Structural formula of (**a**) a 1-butyl-3-methylimidazolium cation [BMIm]^+^, as well as (**b**) bis(trifluoromethylsulfonyl)imide [NTf_2_]^−^, (**c**) trifluoromethanesulfonate [OTf]^−^, and (**d**) dicyanamide [DCA]^−^ anions.

The structure of the cation and anions in the IL under study is shown in Figure 1.

These ionic liquids were chosen because of their high long-time thermostubility, conductivity, and low viscosity, as well as their different hydrophobicity, structure, and size of anions [23,24,25]. Along with strong electrostatic interactions, these compounds can form H-bonds through various electron-donating atoms, such as N, O, and F, in the anion and electron-accepting H atoms in the cation [26]. Moreover, these salts are of interest for use in electrochemical technologies and electrochemical devices [27,28], due to their low corrosivity [29]. The thickened ionic liquids are promising for use in solid-state devices [30] 

The main purpose of the present research was to identify changes in the properties of composites as a result of MMT K10 modification with the listed IL. For this, the structure and morphology of the obtained samples were characterized using different techniques, such as X-ray diffraction (XRD), FTIR, and scanning electron microscopy (SEM). The effect of clay on the thermodynamic properties of ionic liquids was also studied.

## 2. Materials and Methods

### 2.1. Materials

The montmorillonite K10 (Acros organics, Catalog No. AC456071000, Pittsburgh, PA, USA) was used without additional processing. MMT K10 is an acid-activated and thermally treated montmorillonite. During the acidic treatment, the crystal structure of the montmorillonite becomes partially destroyed, which leads to the formation of a highly porous substance with a larger surface area and nanopores. As a result of the thermal treatment, MMT K10 almost loses its ability to swell [30,31].

The ionic liquids of 1-butyl-3-methyl-imidazolium bis (trifluoromethylsulfonyl) imide (Sigma-Aldrich, CAS Number: 174899-83-3, for synthesis, Saint Louis, MO, USA), 1-butyl-3-methylimidazolium trifluoromethanesulfonate (Sigma-Aldrich, CAS Number: 74899-66-2, for synthesis, Saint Louis, MO, USA), and 1-butyl-3-methylimidazolium dicyanamide (Sigma-Aldrich, CAS Number: 448245-52-1, purity ≥97%, Hong Kong, China) were used without further purification.

The MMT K10/IL composites were obtained by direct mixing of the components with a vibration shaker IKA VORTEX 4 Basic (IKA-Werke GmbH & Co, Breisgau, Germany). The mixture was kept in a vacuum drying oven LT-VO/20 (Labtex, Moscow, Russia) at a temperature of 80 °C for 8 h. The obtained mechanical dispersions of the clay mineral particles in the IL were additionally treated in hermetically sealed capsules in an ultrasonic bath CT-431D2 (CTbrand Wahluen Electronic TOOL Co. Ltd., Hong Kong, China) for 8 h. The obtained mixtures had the molar ratio of the IL:MMT K10 components as 2:1, which corresponded to 53.5 wt % of BMImDCA, 61.6 wt % of BMImOTf, and 69.9 wt % of BMImNTf_2_ in the obtained ionogels.

### 2.2. Methods and Apparatus

#### 2.2.1. Dynamic Light scattering (DLS)

Dynamic light scattering (DLS) (Malvern Zetasizer Nano ZS, Malvern, UK) was used to monitor the particle size (radius) in the 0.2–5000 nm range. Before testing, a suspension of clay in distilled water was prepared, sonicated, and kept for 24 h.

#### 2.2.2. Scanning Electron Microscopy (SEM)

The surface morphology and composition of the ММТ K10 powder and MMT K10 samples modified by an ionic liquid were investigated using a Tescan Vega 3 SBH (TESCAN, Brno, the Czech Republic) scanning electron microscope equipped with an energy-dispersive X-ray spectroscopy (EDX) detector. For testing, the ММТ K10 and MMT K10/IL samples were placed on carbon tape. Then a layer of carbon 1 nm thick was applied to the samples. The MMT K10 particle sizes were determined using VEGA3 Control software.

#### 2.2.3. Nitrogen Vapor Adsorption–Desorption

The ММТ K10 porosity quantitative parameters were determined by the method of low-temperature (77 K) nitrogen vapor adsorption–desorption with a Quanta Chrome Nova 1200 (Quantachrome Corporation, Boynton Beach, FL, USA) surface area analyzer. Before the adsorption measurements, the powder had been degassed at 90 °C for 7 h. The isotherms were analysed using the Brunauer-Emmett-Teller (BET) and Barrett-Joyner-Halenda (BJH) models.

#### 2.2.4. X-Ray Diffraction (XRD)

The crystal structure of the ММТ K10 powder and MMT K10 samples modified by an ionic liquid was investigated by X-ray diffraction in the angle range of 2θ = 5°–50° using a DRON-UM1 (Russia) diffractometer (Cu*K*_α_ radiation, λ = 0.154 nm), operating at a voltage of 40 kV and a current of 40 mA.

#### 2.2.5. Fourier-Transform Infrared (FTIR) Spectroscopy

A VERTEX 80v infrared-Fourier spectrometer (Bruker, Karlsruhe, Germany) was used for the spectrophotometric measurements. The FTIR reflection spectra were recorded in the region from 400 to 4000 cm^−1^ at room temperature, and the resolution was 2 cm^−1^. The measurements were carried out in a thin layer of IL deposited on a diamond crystal, by casting at ambient temperature.

#### 2.2.6. Thermogravimetric Measurements

Thermogravimetric analysis was performed using a TG 209 F1 thermal analyzer (NETZSCH, Selb, Germany). The samples of about 10 mg in a platinum crucible were heated in an argon flow (30 mL/min) at a rate of 10 °C/min to a temperature of 600–800 °C, and the weight loss was measured. The accuracy of the sample mass measurement was 1 × 10^−6^ g, and the accuracy of the temperature measurement was 0.1 °C. Three TG scans were performed for each sample.

#### 2.2.7. Differential Scanning Calorimetry (DSC)

A DSC 204 F1 Phoenix calorimeter (NETZSCH, Selb, Germany) was used to determine the thermodynamic parameters, such as the melting point (T_m_), crystallization (T_c_), glass transition temperature (T_g_), and the heat capacity change (Δ*C*_p_) of phase transition. The sample of approximately 10 mg in a hermetically sealed platinum pan was heated to 80 °C, cooled with liquid nitrogen to −110 °C, and then heated to 150 °C. The rate of both cooling and heating was 10 °C/min. The measurements were carried out in an argon atmosphere. The accuracy of the temperature measuring was ±0.1 °C.

## 3. Results and Discussion

### 3.1. Characterization of Montmorillonite K10 Powder

**Figure 2:**
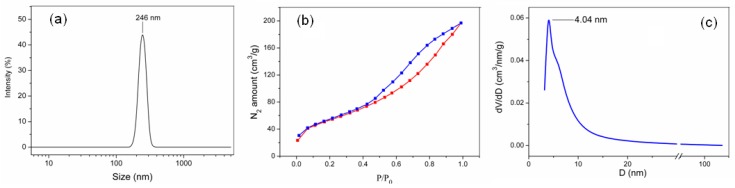
Characterization of montmorillonite (MMT) K10 powder: (**a**) Size distribution by intensity (dynamic light scattering (DLS)) particles; (**b**) isotherms of low-temperature nitrogen adsorption-desorption on particles; (**c**) Barrett-Joyner-Halenda (BJH) pore size distribution for MMT K10.

The results of the MMT K10 particle size distribution, obtained using DLS, are presented in the form of a histogram in Figure 2a. As shown, the sizes of most of the particles lie within the range from 200 to 300 nm. The particle size distribution is monomodal, with a peak at 246 nm.

In order to determine the structural and adsorption parameters of the MMT K10 powder, the nitrogen adsorption/desorption isotherm was recorded. Figure 2b shows that the nitrogen adsorption/desorption isotherm belongs to type IV, according to the IUPAC classification [32]. The following parameters were determined by this technique: the specific surface area of the MMT K10 according to BET, which is 195 m^2^/g; the total pore volume, which is 0.305 cm^3^/g; and the average pore size, which is 6.25 nm.

Figure 2c shows the pore size distribution curve obtained using the BJH model. We can see that 80% of the total pore volume is pores with a diameter smaller than 25 nm. This provides evidence for the framework mesoporosity of the clay. It can be seen that the pore size distribution is monomodal, with a peak at 4.04 nm.

**Figure 3:**
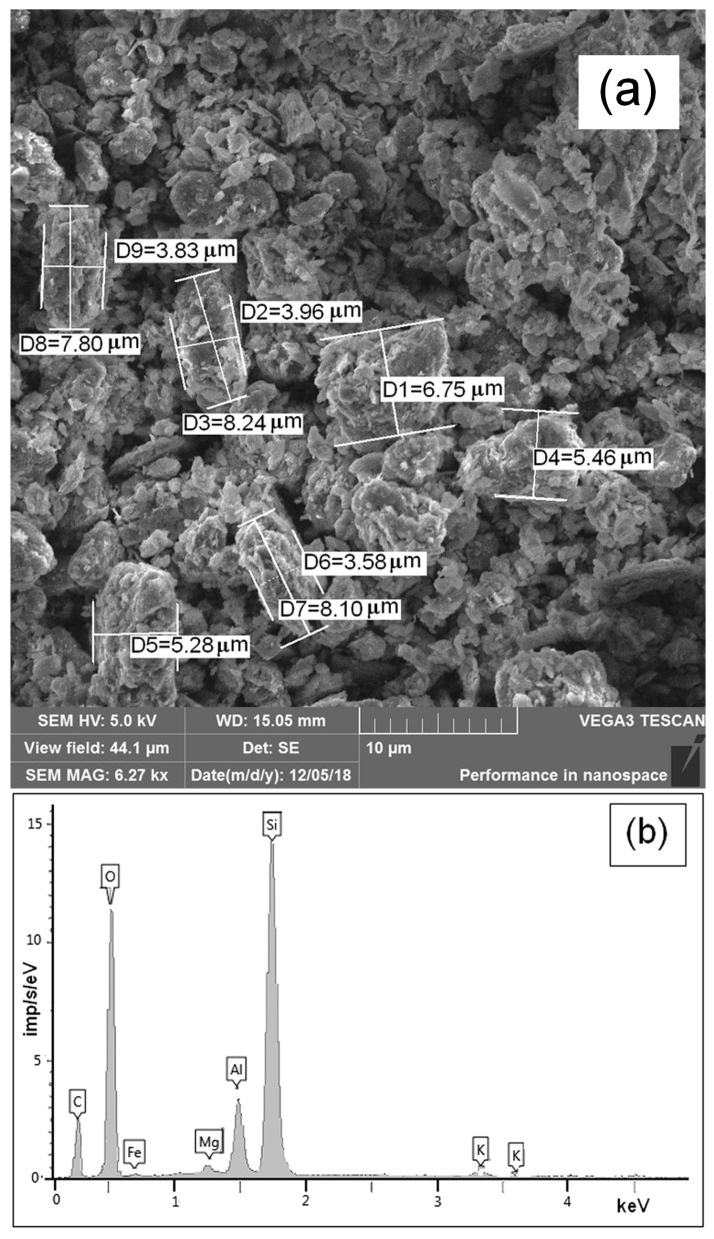
Scanning electron microscope (SEM) image (**a**) and energy-dispersive X-ray spectroscopy (EDX) analysis (**b**) of the MMT K10.

The scanning electron microscopic technique was used to explore the surface morphology of both MMT K10 and MMT K10/IL composite. The clay samples have the shape of polydisperse particles 150–300 nm in size, in the form of agglomerates (Figure 3a). These results agree with the data obtained by the DLS method.

An EDX analysis was also performed (Figure 3b). The main elements in pure MMT K10 are oxygen (about 62.85%) and silicon (about 27.66%). At the same time, the clay spectrum contains Al (5.81%), K (0.63%), and Mg (0.80%) elements.

### 3.2. Structure and Properties of the Montmorillonite K10/IL Composite Materials

#### 3.2.1. Surface Morphology and Composition

**Figure 4:**
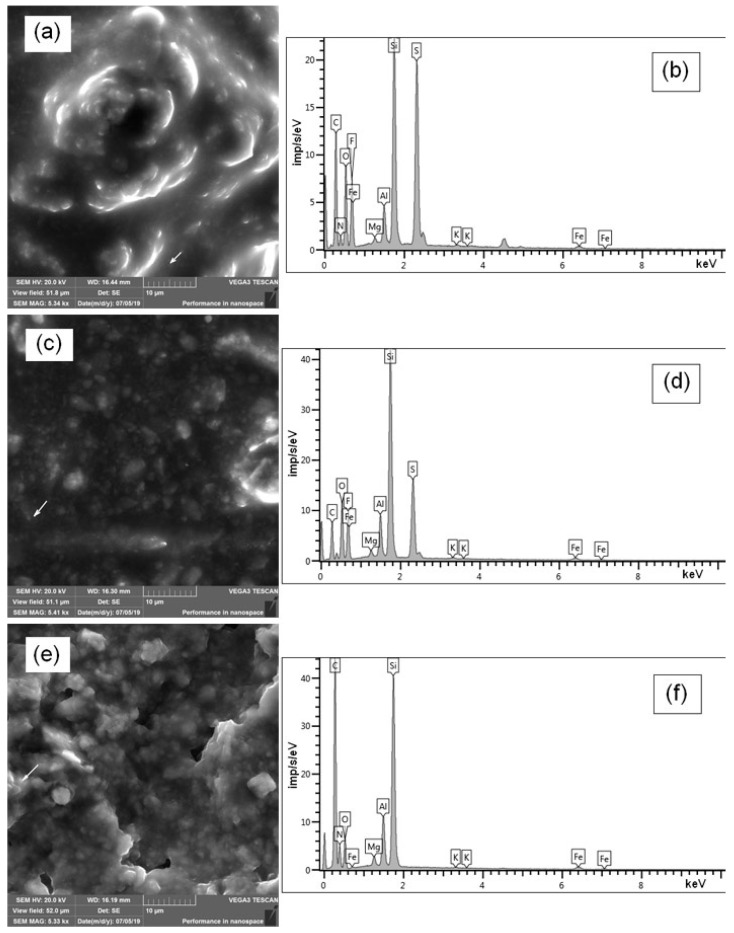
SEM-images (left) and EDX analysis (right) of the samples of MMT K10/BMImOTf (**a**,**b**), MMT K10/BMImDCA (**c**,**d**), and MMT K10/BMImNTf_2_ (**e**,**f**).

The surface morphology of the MMT K10/IL composites was researched using SEM and is presented in Figure 4a,c,e. It can be seen that the MMT K10/IL composite shows a massive, thinly layered structure, with some interlayer spaces and large flakes. This may be attributed to the chemical modification by large alkyl moieties in the clay galleries. Thus, the MMT K10/IL composite is an ionic liquid filled with aggregated montmorillonite particles, which play the role of thickeners, and the ionic liquid completely surrounds the clay particles.

Figure 4b,d,f shows the results of the EDX analysis for the MMT K10/IL under study. In addition to oxygen and silicon as the main elements of MMT K10, these composites contain sulfur, fluorine, and nitrogen, which are component parts of ionic liquids.

#### 3.2.2. Crystal Structure of the Montmorillonite K10/IL Composites

**Figure 5:**
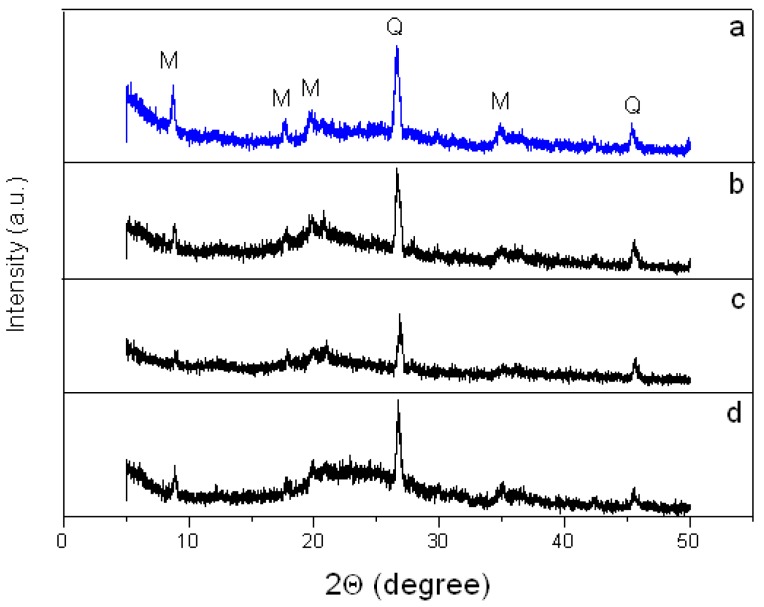
X-ray diffraction (XRD) patterns for MMT K10 (**a**), MMT K10/BMImOTf (**b**), MMT K10/BMImNTf_2_ (**c**), and MMT K10/BMImDCA (**d**).

Figure 5a–d shows the diffraction patterns of X-ray scattering for the MMT K10 and MMT K10/IL composites. These patterns contain reflections corresponding to ММТ ((001), (002), (110), (220)) and quartz ((101), (112)). The enumerated peaks are characteristic of both the original clay and the materials containing an IL. In addition, the introduction of ILs leads to minimal changes in the crystal structure of the clay. This is confirmed by the data in Table 1. Regardless of the IL’s nature, during the composite formation, the reflexes are shifted by no more than 0.3 degrees, and the interlayer distance (*d*) in the MMT K10, calculated by Bragg’s law, changes by no more than 0.015 nm.

As Table 1 shows, in the studied materials, the value *d_001_* is equal to about 1 nm. This value is a sum of the thickness of the silicate plate (0.96 nm) and the gallery thickness [33]. Taking into account that the diameter of the [BMIm]^+^ cation is 0.66 nm [34], it can be concluded that the IL is mostly adsorbed on the clay surface. Evidently, the ionic liquid promotes partial dehydration of the interlayer space. It should be also said that this supposition agrees with the TG analysis and FTIR spectroscopy results described below.

Note that position of the (001) reflection for MMT K10 is discussed in publications. Also, different researchers come to different conclusions. For instance, the authors of [35] did not observe this peak in montmorillonite K10. They explained that this may be due to the damage to the clay layers caused during acid treatment, but the reflection observed at 9° creating doubt about the (001) peak is due to the illite impurity present in the clay. Other researchers observed in MMT K10 a wide (001) reflection at lower angles (about 5.8°) [36]. According to them, this peak corresponds to remaining montmorillonite, reflecting a quite disordered stacking in the layers. Therefore, it is difficult to draw final conclusions about changes in the crystalline structure of clay with the introduction of IL.

In addition, we calculated the crystallite size using the XRD data and Scherrer’s formula. We found the montmorillonite K10 plate width was 34.6 nm. This size is smaller than the particle size of montmorillonite, according to SEM (Figure 3a). Apparently, montmorillonite particles contain many crystallites. Therefore, in our opinion, it is not entirely correct to compare the particle sizes obtained using XRD and SEM.

#### 3.2.3. Phase Transitions in the Montmorillonite K10/IL Composites

**Figure 6:**
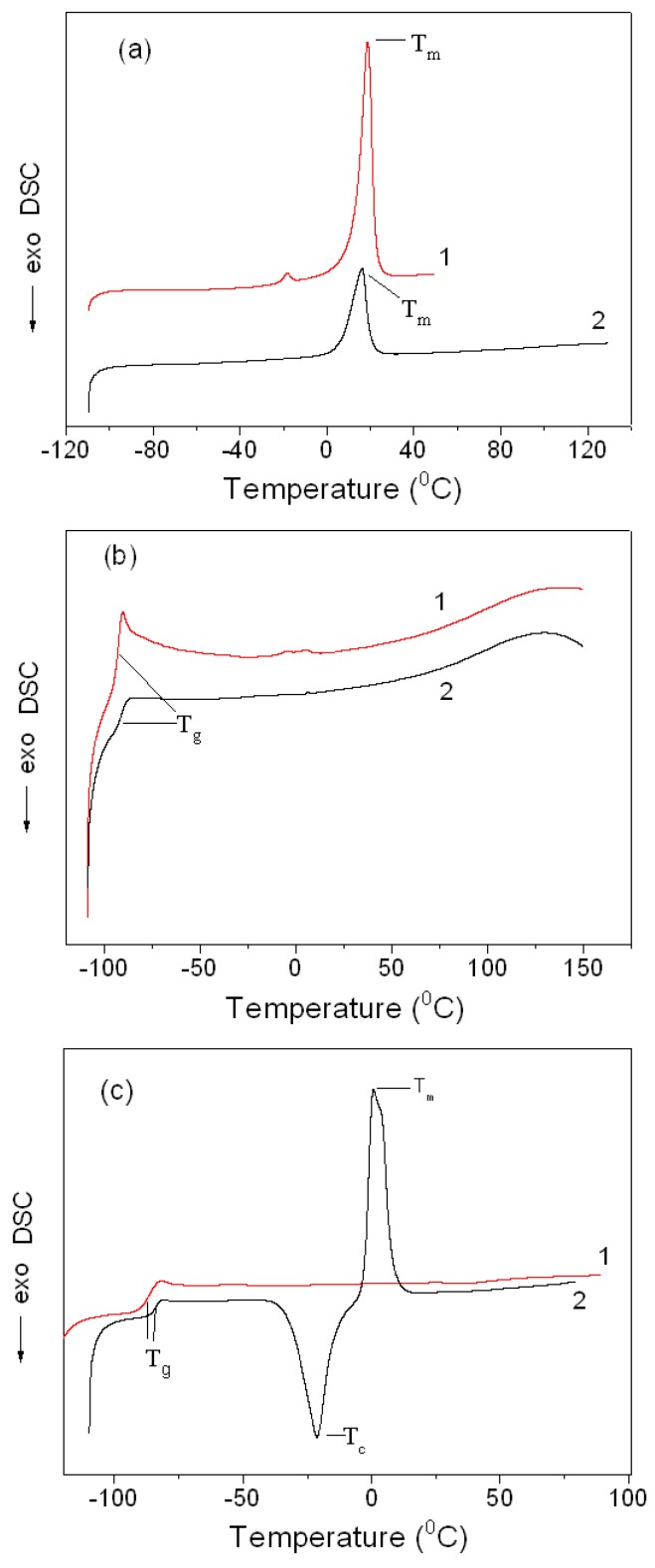
Differential scanning calorimetry (DSC) curves of the second heating cycle (**a**) 1: BMImOTf, 2: MMT K10/BMImOTf; (**b**) 1: BMImNTf_2_, 2: MMT K10/BMImNTf_2_; (**c**) 1: BMImDCA, 2: MMT K10/BMImDCA.

Figure 6a–c show thermograms of the second heating cycles for the studied ILs and MMT K10/IL composites recorded by the DSC method in the temperature range from −130 to +280 °C. In these thermograms, the inflection point of the curve corresponds to the glass transition temperature, the exothermic peak (*T_c_*) corresponds to crystallization, and endothermic peak (*T_m_*) corresponds to melting.

As Figure 6a shows, when the BMImOTf and composites containing this ionic liquid are heated, the thermograms have an endothermic peak corresponding to melting. Other phase transitions are not observed. The characteristic temperatures of the thermal behaviour of the ionic liquid and its composite with the clay are given in Table 2.

The thermal behaviour of the BMImDCA and ММТ K10/BMImDCA composites are different. As Figure 6b shows, there is a phase transition caused by glass transition processes in the low-temperature regions (−90–−95 °C) of such systems. It should be said that the specific heat change (Δ*C*_p_) in the ММТ/BMImDCA composite is much smaller than in the pure IL: ≈0.2 J/(g·K) for the composite and ≈0.6 J/(g·K) for BMImDCA.

Figure 6c shows DSC curves for BMImTf_2_N and the MMT K10/BMImNTf_2_ composite. As it indicates, in this case, the thermogram has an inflection point corresponding to *T_g_*. As Table 2 shows, that clay introduction into the IL increases the glass transition temperature of BMImNTf_2_ by 3 °C. This inflection is observed both in the first and the second heating cycles. In addition, heating of the MMT K10/BMImNTf_2_ composite leads to the appearance of a broad exothermic peak of crystallization, and then to an endothermic peak of melting. However, in case of the IL, there are no crystallization or melting peaks in the second heating cycle. It should be said that for BMImNTf_2_, the characteristic temperatures found in the first heating are close to the data reported previously [37].

Thus, when the IL is adsorbed on the clay surface, the phase transitions in the IL change. The greatest changes are observed in the case of BMImNTf_2_, which is consistent with the data of the FTIR spectroscopy described below.

#### 3.2.4. Thermal Degradation of the Montmorillonite K10/IL Composites

**Figure 7:**
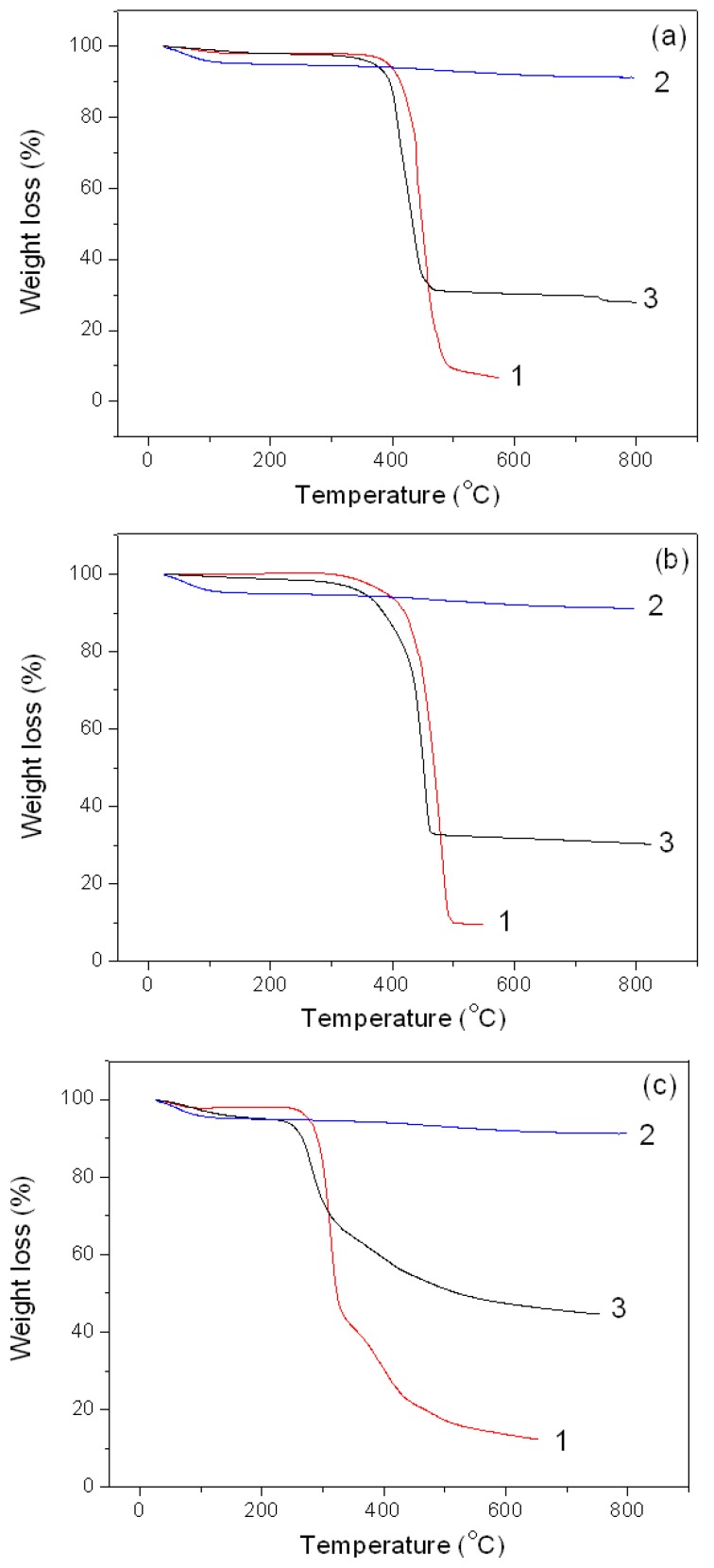
TG curves: (**a**) 1: BMImOTf, 2: MMT K10, and 3: MMT K10/BMImOTf; (**b**) 1: BMImNTf_2_, 2: MMT K10, and 3: MMT K10/BMImNTf_2_; (**c**) 1: BMImDCA, 2: MMT K10, and 3: MMT K10/BMImDCA.

Figure 7a–c show TG curves recorded for the studied ILs and MMT K10/IL composites. It can be seen that the thermal degradation of the studied materials proceeds in several stages. The onset and end temperatures (*T*_1_ and *T*_2_) and the weight loss (Δm_i_) of each of the stage, as well as temperature corresponding to the maximum thermal decomposition rate (*T_d_*) were determined and are presented in Table 3.

At the first stage, the mass reduction in the samples (Δm_1_) did not exceed 5%, and evidently, it was caused by the water removal. It was also accompanied by a considerable widening of the temperature range (by 40–50 °C) of the first stage for the composites, in comparison with the individual ILs. This effect may be due to the interaction of IL with clay. Comparing the Δm_1_ values for the composites studied (Table 3), we can conclude that this value is maximum for MMT K10/BMImDCA. Therefore, the origin of the anion in the IL affects the intensity of water removal from the composite.

The second stage is characterized by a sharp change in the mass of the samples, and is associated with the IL thermolysis itself. At this stage, the Δm_2_ values exceed 90% for the individual BMImOTf and BMImNTf_2_, as well as about 68% for their composites. It should be noted that for the samples based on BMImOTf and BMImNTf_2_, further heating (to 800 °C) does not result in thermal decomposition of the samples, which is associated with the clay stability in the composites.

Figure 7c shows that the thermal behaviour of materials based on DCA is different. For these materials, the decomposition thermograms have a third stage, at which there occurs a slow mass loss. It is associated with the degradation of the [BMIm]^+^ cation, which is more stable than the [DCA]^−^ anion [38,39].

It is worth mentioning that at the transition from the IL to the MMT K10/IL composite, values of *T*_1_, *T_d_*, and *T*_2_ decrease considerably, which is reflected in the shift of the TG curves to the region of lower temperatures. This means that the introduction of the IL to the composite with MMT K10 reduces its thermal stability. This effect is observed regardless of the nature of the studied ionic liquid, and is evidently associated with the weakening of the ionic liquid cation and anion bonds, as well as the formation of intermolecular bonds with the active groups of the alumosilicate, which is confirmed by the FTIR data discussed below.

Therefore, when interacting with clay, the thermal behaviour of the ionic liquid changes significantly. This may be used to regulate the properties of materials based on IL.

#### 3.2.5. FTIR Spectra of the Montmorillonite K10/IL Composites

**Figure 8:**
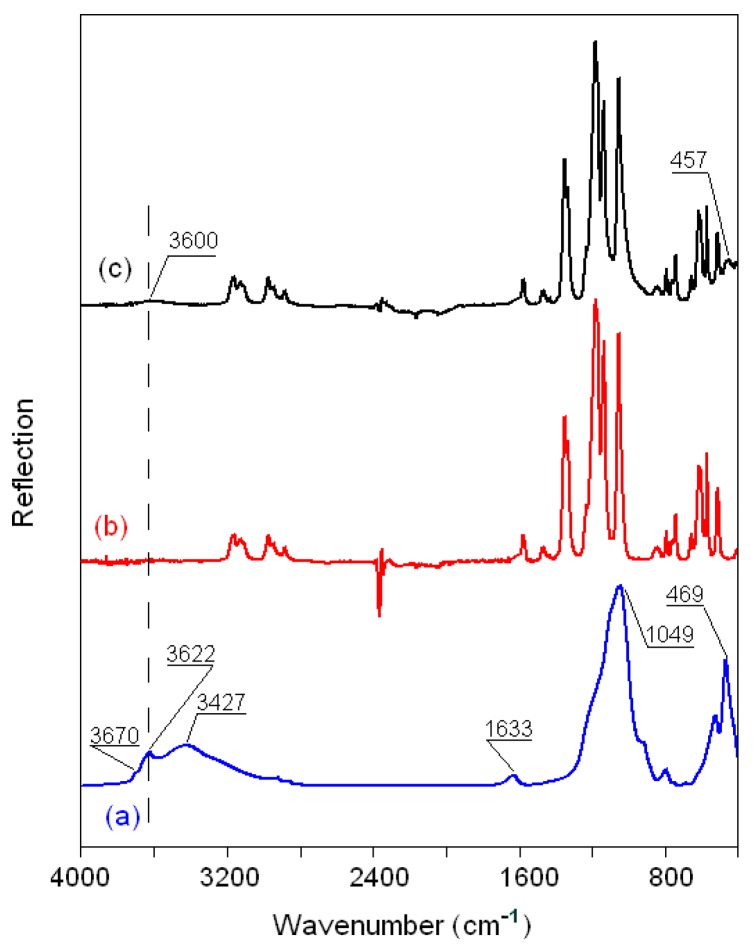
FTIR spectra of (**a**) MMT K10, (**b**) BMImNTf_2_, and (**c**) MMT K10/BMImNTf_2._

**Figure 9:**
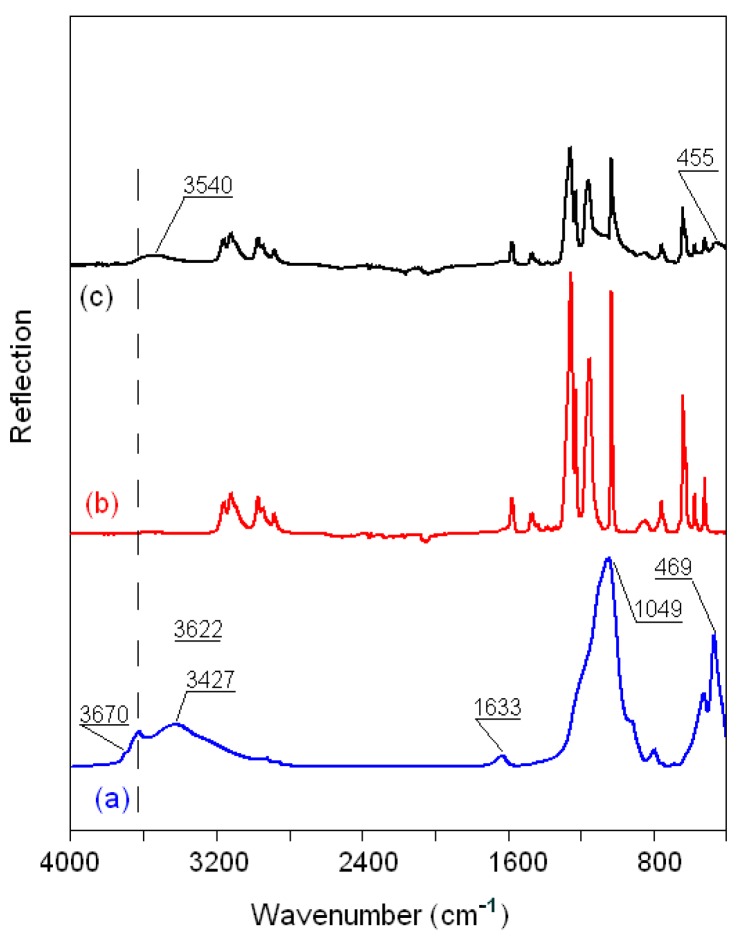
FTIR spectra of (**a**) MMT K10, (**b**) BMImOTf, and (**c**) MMT K10/BMImOTf.

**Figure 10:**
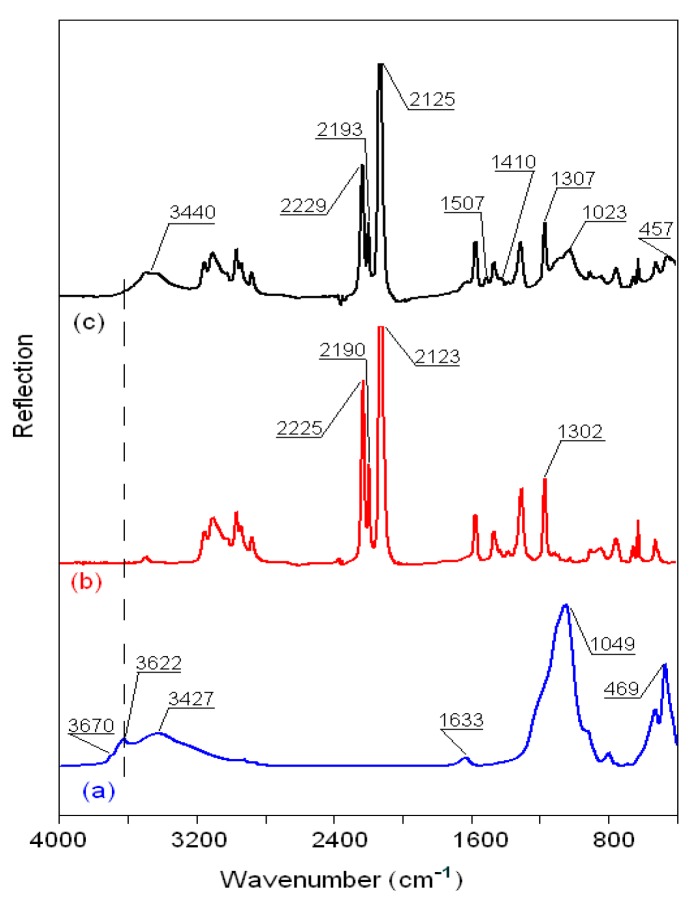
FTIR spectra of (**a**) MMT K10, (**b**) BMImDCA, and (**c**) MMT K10/MImDCA.

The FTIR reflection spectra of the original montmorillonite K10 powder; pure ionic liquids BMImNTf_2_, BMImOTf, and BMImDCA; and MMT K10 samples modified with ionic liquids are compared in Figure 8, Figure 9 and Figure 10 in the region of 4000–400 cm^−1^. The bands of MMT K10 at 3622 and 3670 cm^−1^ (a shoulder; trace (a) in Figure 8, Figure 9 and Figure 10) correspond to the interlayer hydroxyl stretching (*ν*Si–OH) [40,41]. The broad band at ca. 3427 cm^−1^ and the minor peak at 1633 cm^−1^ have been assigned to the stretching (*ν*OH) and bending (δOH) modes of absorbed water. The silicon–oxygen (*ν*SiO) and aluminum–oxygen (*ν*AlO) bonds are observed at 1049 cm^−1^ and 620 cm^−1^ (a shoulder), and the magnesium–oxygen (*ν*MgO) bond is assigned to the band between 470 and 530 cm^−1^ [41,42]. The assignments of the important bands of the pure MMT K10 clay and pure ionic liquids are listed in Table 4, according to [40,41,42,43,44,45].

The FTIR spectra of all the modified MMT K10 are quite similar to those of the ionic liquids. In the MMT K10/BMImNTf_2_ spectrum, the *ν*OH band at 3427 cm^−1^ from MMT K10 is observed in the form of a small wave centered at 3600 cm^−1^ (trace (c) in Figure 8), which indicates that the addition of BMImNTf_2_ leads to a decrease in bound water. The peaks at 2800–3200 cm^−1^ from the BMImNTf_2_ ionic liquid are practically unaffected, since their displacements do not exceed the instrumental resolution (2 cm^−1^). The spectrum from 1300 to 800 cm^−1^ is a superposition of bands from the ionic liquid and clay, due to oxygen-containing groups (Table 4), and therefore cannot provide much information. However, in the range of 600–400 cm^−1^, there is one peak from MMT K10, which changes in comparison with pure clay. The same trend is observed in the spectrum of MMT K10/BMImOTf, but the *ν*OH peak appears at a lower frequency, about 3540 cm^−1^ (trace (c) in Figure 9), which indicates that water is associated with an ionic liquid (mainly with the [OTf]^−^ anion) [46].

In the MMT K10/BMImDCA spectrum, the wave from *ν*OH modes is already at 3440 cm^−1^, and the *ν*SiO mode of pure MMT K10 (1049 cm^−1^) is clearly observed and shifted from 1049 in pure clay to 1023 cm^−^ (trace (c) in Figure 10). In addition, more information can be observed in the frequency range of the cation (2300–2050 cm^−1^) and anion (1600–1300 cm^−1^). For example, for the [BMIm]^+^ cation, new bands are observed at 1507 and 1410 cm^−1^, and the peaks at 1302 cm^−1^ (δ_as_^ip^ ring and δCCCC modes) and 2123–2225 cm^−1^ (*ν*_s_(N≡C) mode) show blue shifts of about 2–4 cm^−1^. It has also been found that the ν(Mg–O) mode of pure MMT K10 shows a red shift (∆) to the lower frequency range in the spectra of all the modified MMT K10, the ∆ν(Mg–O) values are 12, 14, and 12 cm^−1^ for the MMT K10/BMImNTf_2_, MMT K10/BMImOTf, and MMT K10/BMImDCA samples, respectively.

Thus, the characteristic frequencies of the initial components have noticeable multidirectional shifts, caused by the replacement of water molecules adsorbed by the clay with molecules of the added salt, followed by the interaction of ionic liquid with both clay and water. The interaction of water and clay with confined ionic liquid changes in the series [NTf_2_]^−^ < [Otf]^−^ < [DCA]^−^, in accordance with the decrease in hydrophilicity of the anion in the series [DCA]^−^ > [Otf]^−^ > [NTf_2_]^−^, and in accordance with an increase in the size of the anion in the series [DCA]^−^ < [OTf]^−^ < [NTf_2_]^−^. The interaction of montmorillonite K10 with ionic liquids occurs mainly due to electrostatic bonds of the cation with the silane group SiO (as well as with MgO) of clay. It can be assumed that the dicyanamide anion, due to its small size, also interacts with the silanol group through the hydrogen bond Si–OH····N.

In summary, we can say the following. The melting point and thermal stability determine the range of the liquid state of an IL, and therefore, the area of their application. For clay-thickened ionic liquids, it is important that the temperature range in which the IL exhibits the properties of a low-temperature melt expands compared to pure IL, and that the decrease in melting point and thermal stability are insignificant. This allows the use of IL as part of solid-state devices without loss of quality.

Both the melting point and thermal stability of ILs are largely dependent on intramolecular and intermolecular interactions. Coulomb forces between the anion and the cation, van der Waals bonds between alkyl chains of imidazolium rings, and H-bonds are the most important interactions in ionic liquids. Therefore, the melting point and thermal stability of ILs depend on the type of both the cation and the anion. At the same time, for IL–clay composites, these parameters will also depend on the interaction of IL with the clay surface. To determine the effect of anion types of ILs on the thermal behaviour of nanocomposites, the interaction of an IL based on 1-butyl-3-methylimidazole with montmorillonite K10 was first studied.

Montmorillonite K10 is acid-treated clay with a highly developed porous structure, which has a significant adsorption capacity and high catalytic activity in various reactions of organic synthesis. It was found that the interaction of the IL with MMT K10 occurs due to the processes of desorption of water molecules from the clay surface and the adsorption of the IL’s molecules; in addition, the confined ionic liquid interacts with both clay and water. Hydrophobic IL partially replaces water, while hydrophilic IL is introduced into a layer of water adsorbed on the surface of the clay. These processes depend on the nature of the anion, and lead to noticeable multidirectional shifts of the characteristic frequencies in the IR spectra of the original components. It was found that the interaction of confined ionic liquid with MMT K10 and adsorbed water changes in the following order: [DCA]^−^ > [OTf]^−^ > [NTf_2_]^−^, in accordance with the increase in hydrophilicity and decrease in the size of the anion. MMT K10 interacts with ionic liquids mainly due to electrostatic and van der Waals bonds of the cation with the silane groups of clay. The anion dicyanamide apparently also interacts with silanol groups through hydrogen bonds.

Based on these data, it can be assumed that the decrease in thermal stability of confined ILs is related to their interaction with the surface of MMT K10, as well as changes in the hydrate state of the clay resulting from interaction with the ionic liquid. This is clearly demonstrated by the TG data. Heating nanocomposites based on hydrophobic ILs MMT K10/BMImOTf and MMT K10/BMImNTf_2_ does not lead to mass change, due to the removal of adsorbed water. On the contrary, heating the MMT K10/BMImDCA composite with a hydrophilic ionic liquid is accompanied by a significant decrease in mass due to the removal of H_2_O. At the same time, the thermal stability of ILs decreases by about 30 °C. Thus, the highly developed surface of the acid-treated MMT K10 contributes to the thermal decomposition of the adsorbed IL.

## 4. Conclusions

In this work, composites based on montmorillonite K10 and ionic liquid 1-butyl-3-methylimidazole with various anions, such as bis (trifluoromethylsulfonyl) imide, trifluoromethanesulfonate, and dicyanamide were studied for the first time. The thermal behaviour of the MMT K10/IL composite and the corresponding IL have been compared by the DSC and TG methods. It has been identified that the anion nature in the IL affects phase transitions in the materials in question. It has also been found that pure ILs have higher (by 30–40 °C) characteristic temperatures of thermal decomposition compared to the corresponding MMT K10/IL composites. Based on the data obtained by the XRD and FTIR methods, it has been concluded that the presence of an IL leads to water removal from the interlayer space in the clay. The interaction of IL and MMT K10 depends on the nature of the ionic liquid anion. Molecules of the ionic liquids are immobilized mainly on the surface of the clay, and interact with molecules of adsorbed water.

## Figures and Tables

**Table 1:** Results of X-ray spectral analysis for MMT K10 and MMT K10/IL composites.

Reflection Index	MMT K10		MMT/BMImOTf		MMT/BMImNTf_2_		MMT/BMImDCA	
	2θ, deg	*d*, nm	2θ, deg	*d*, nm	2θ, deg	*d*, nm	2θ, deg	*d*, nm
(001)	8.74	1.011	8.88	0.995	8.91	0.992	8.87	0.996
(002)	17.67	0.502	17.84	0.497	17.87	0.496	17.76	0.499
(110)	19.65	0.451	19.64	0.452	19.85	0.447	19.95	0.445
(101)	26.54	0.336	26.60	0.335	26.84	0.332	26.76	0.333
(220)	34.85	0.257	34.94	0.257	35.0	0.256	34.96	0.256
(112)	45.38	0.200	45.55	0.199	45.68	0.198	45.53	0.199

**Table 2:** Characteristic temperatures of thermal behaviour of the samples under study, determined by DSC.

Sample	*T_g_*, °C	*T_c_*, °C	*T_m_*, °C
BMImOTf			18.7
MMT K10/BMImOTf			16.2
BMIm DCA	−92.7		
MMT K10/BMImDCA	−91.6		
BMImNTf_2_	−86.3–−86.8 ^a^	−48.6 ^b^–−46.3 ^a,b^	−6.5 ^b^–−6.5 ^a,b^
MMT K10/BMImNTf_2_	−83.5	−19.3	0.7

a. Reported in [37]; b. The onset temperature of exothermic (*T_c_*) and endothermic (*T_m_*) peaks detected in the first heating cycle.

**Table 3:** Characteristic temperatures (*T*) and mass loss (Δm_i_) of the thermal decomposition of the samples under study.

Parameter	BMImOTf	MMT K10/BMImOTf	BMImNTf	MMT K10/BMImNTf_2_	BMImDCA	MMT K10/BMImDCA
First stage						
*T_1_*, °C	54.3	61.9	-	53.3	40.1	52.2
*T_2_*, °C	110.1	164.2	-	119.0	80.6	130.2
Δm_1_, %	1.9	2.1	-	1.3	1,9	4.9
Second stage						
*T_1_*, °C	427.7	392.8	441.1	425.2	293.7	260.7
*T_d_*, °C	440.9	408.9	481.5	453.8	313.5	280.4
*T_2_*, °C	462.8	449.5	500.1	460.4	324.1	303.7
Δm_2_, %	91.6	67.7	90.4	68.6	57.4	30.9
Third stage						
*T_1_*, °C	-	-	-	-	372.1	374.9
*T_d_*, °C	-	-	-	-	393.0	385.8
*T_2_*, °C	-	-	-	-	428.4	482.0
Δm_3_, %	-	-	-	-	28.4	20.1

**Table 4:** Some important peaks in the FTIR spectra of pure ionic liquids BMImNTf_2_, BMImOTf, and BMImDCA, as well as pure MMT K10 clay, and their assignments.

Wavenumber (cm^−1^)	Assignment [40,41,42,43,44,45]
*Clay* 3630–3700	*ν*Si–OH
3300–3500	*ν*OH (H_2_O)
1633	δOH (H_2_O)
1049	*ν*SiO (Si–O–Si)
620	*ν*AlO (Al–O–Al)
529–469	*ν*MgO (Mg–O–Mg)
*Ionic liquid*	
3000–32000	ν_as_H(C4,5)H, *ν*_s_H(C4,5)H, *ν*C2H
2800–3000	*ν*CH_3_, *ν*CH_2_, *ν*NH
2100–2300	*ν*_s_(N≡C)
1150–1600 ^a^	νCH_2_(N), νCH_3_(N), ring CH_3_ and νCN, νCCCC, δ_as_^ip^ ring and δCCCC, δCH_3_(N)CN
1000–1200 ^b^	*ν*_s_SO_3_, *ν*_s_CF_3_
1100–1200 ^b^	*ν*_as_CF_3_
1000–1050 ^b^	*ν*_s_SO_3_
1300-137 °C	*ν*_as_SO_2_
1110-125 °C	*ν*_s_CF_3_, *ν*_as_CF_3_, *ν*_s_SO_2_
1000-180 °C	*ν*_a_SNS

a. Peaks observed in the BMImDCA spectrum; b. peaks observed in the BMImOTf spectrum; ^c^ peaks observed in theBMImNTf_2_ spectrum; *ν*_s_ and *ν*_as_: symmetric and asymmetric stretching, respectively; δ_s_ and δ_as_: symmetric and asymmetric bending, respectively.
